# Intelligent Control System for Brain-Controlled Mobile Robot Using Self-Learning Neuro-Fuzzy Approach

**DOI:** 10.3390/s24185875

**Published:** 2024-09-10

**Authors:** Zahid Razzaq, Nihad Brahimi, Hafiz Zia Ur Rehman, Zeashan Hameed Khan

**Affiliations:** 1 Faculty of Engineering, Free University of Bozen-Bolzano, 39100 Bozen-Bolzano, Italy; 2 Department of Informatics, Bioengineering, Robotics and Systems Engineering (DIBRIS), University of Genoa, 16126 Genova, Italy; 3 School of Computer Science and Technology, Beijing Institute of Technology, Beijing 100081, China; brahiminihad@bit.edu.cn; 4 Department of Mechatronics Engineering, Air University, Islamabad 44000, Pakistan; 5 Interdisciplinary Research Center for Intelligent Manufacturing and Robotics (IRC-IMR), King Fahd University of Petroleum and Minerals, Dhahran 31261, Saudi Arabia; zeashan.khan@kfupm.edu.sa

**Keywords:** brain-computer interface, neuro-fuzzy control, shared control, self-learning, mobile robots, intelligent control, fuzzy logic, EMOTIV EPOC+

## Abstract

Brain-computer interface (BCI) provides direct communication and control between the human brain and physical devices. It is achieved by converting EEG signals into control commands. Such interfaces have significantly improved the lives of disabled individuals suffering from neurological disorders—such as stroke, amyotrophic lateral sclerosis (ALS), and spinal cord injury—by extending their movement range and thereby promoting self-independence. Brain-controlled mobile robots, however, often face challenges in safety and control performance due to the inherent limitations of BCIs. This paper proposes a shared control scheme for brain-controlled mobile robots by utilizing fuzzy logic to enhance safety, control performance, and robustness. The proposed scheme is developed by combining a self-learning neuro-fuzzy (SLNF) controller with an obstacle avoidance controller (OAC). The SLNF controller robustly tracks the user’s intentions, and the OAC ensures the safety of the mobile robot following the BCI commands. Furthermore, SLNF is a model-free controller that can learn as well as update its parameters online, diminishing the effect of disturbances. The experimental results prove the efficacy and robustness of the proposed SLNF controller including a higher task completion rate of 94.29% (compared to 79.29%, and 92.86% for Direct BCI and Fuzzy-PID, respectively), a shorter average task completion time of 85.31 s (compared to 92.01 s and 86.16 s for Direct BCI and Fuzzy-PID, respectively), and reduced settling time and overshoot.

## 1. Introduction

Robots are getting popular in healthcare assistance specially for patients with special needs or suffering from restricted movement due to disabilities [1]. The number of people with such disabilities is increasing due to rising cases of stroke, war casualties, road accidents, and aging factors [2,3]. Assistive robots can ameliorate the lives of disabled people; hence, they are growing in demand these days. Usually, healthy individuals can control robots using common input devices—e.g., joystick, keyboard, or mouse. However, it is difficult to operate these devices for individuals with disabilities, such as stroke, amyotrophic lateral sclerosis (ALS), and multiple sclerosis (MS). In most cases, these patients lose the ability to walk, use their hands/arms, or even talk. Thus, disabled individuals cannot easily communicate their intentions to robots using traditional devices [4].

The development of BCIs has helped patients with neurological disorders and disabilities to ameliorate the quality of life [5]. BCI systems infact measure the brain activity and decode their thoughts or intentions into control commands, thus bypassing the peripheral nervous system or muscles [6]. They also play an important part to ensure independence for people with severe disabilities [6,7]. Electroencephalography (EEG) is the most frequently used signal for the development of BCI systems due to its moderate price, easy usage, and high temporal resolution [8]. Furthermore, they can also be used for a variety of tasks: such as 2-D cursor control [9], playing games [10], browsing the Internet [11], brain-controlled vehicles [12], brain-controlled robots and drones [13,14]. The development of an EEG-based brain-controlled robot is a step forward to describe a robot that follows the commands sent by a human brain meant to improve the freedom of movement and quality of life of disabled people [1]. Disabled users can therefore take advantage of a brain-controlled mobile robot such as a wheelchair or electric vehicle (EV) to reach their desired locations with ease and safety using BCIs.

The concept of a BCI mobile robot based on non-invasive EEG signals was first proposed in 2004 by Millán [15]. Afterwards, several researchers developed numerous brain-controlled mobile robots [1,16]. Tanaka [17] presented a BCI robotic wheelchair and tested it in a real-world scenario. In his work, the left and right movements were controlled by motor imagery (MI) generated by the subjects. Rebsamen [18] also developed a BCI robotic wheelchair by using a P300-based BCI to control it from one destination to another.

These studies focused primarily on direct control by BCI, meaning that users issue control commands to steer robots directly. Because the efficiency of BCIs determines the efficiency of these BCI robots, the BCIs can negatively affect their performance: they can come with limitations, such as the number and accuracy of commands and their execution time) [19,20,21]. Consequently, these robots are typically less safe, their performance is currently slow and uncertain, and users feel fatigued when operating for a longer period. Improved BCI techniques can enhance the outcome of BCI systems. However, it is challenging to achieve the desired performance using current BCI techniques because of the non-stationary property of EEG signals and the differences in BCI performance between individuals [1,22,23].

Shared control is a popular approach in human-robot interaction in which humans and machines work together to complete a task by enhancing each other’s capabilities. Shared control techniques can play a part in ameliorating the performance of brain-controlled systems, given the performance constraints of BCIs [24,25,26]. For example, Li [27] proposed a shared-control approach for navigation and control of a wheelchair. In his work, he used the brain-machine control (BMC) mode to produce a polar polynomial trajectory using steady-state visual evoked potentials (SSVEPs) and the autonomous control mode to navigate the robot through the obstacles. Shared control techniques were also proposed by [28,29] using model predictive control (MPC) and sliding mode control (SMC). Alqumsan presented a study on self-adaptations in EEG-based BCIs. The proposed Bayesian update rule can track user goals and benefit the shared control driving scheme by reducing user effort [30]. However, these methods require accurate mathematical models of the system.

A promising model-free approach for developing a shared control system is the fuzzy logic control (FLC) strategy. Fuzzy inference systems (FIS) have been widely used as adaptive controllers for robots [31,32]. A shared control method based on fuzzy logic was proposed by Liu [33]. In her work, the FLC method was used to implement two behaviors (wall following and obstacle avoidance) to navigate and keep the brain-actuated robot safe. Fuzzy-based shared control was also proposed in our previous work [34] for a brain-controlled mobile robot.

The FLC approach has several advantages over traditional control systems, including fuzzy-based control schemes. For instance, it is a model-free method and is robust to disturbances and uncertainties. The fuzzy model is based on data samples using expert experience and simple logic [35,36]; however, it is challenging to design its membership functions, rule base, and tune its parameters. FLC and Artificial Neural Networks (ANN) have been successfully employed to model and control various control problems because they combine the benefits of both the FLC and ANN systems—such as human reasoning and learning capability. Neuro-fuzzy systems have received considerable attention in the literature and have become a rapidly emerging field. The Adaptive Neuro-Fuzzy Network (ANFIS) proposed by Jang [37] is one of the popular neuro-fuzzy method in use. However, it requires offline I/O training data and cannot update its parameters online to cope with changes in plant dynamics or handle disturbances during the process. The Self-Learning Neuro-Fuzzy (SLNF) controller [38] used in our study can address this problem. The Feedback error learning (FEL) scheme, first proposed by Kawato [39], is used in this study to train the SLNF controller. It was found that the FEL control technique provides excellent tracking performance without substantial modeling, which is desirable in our work. The uniqueness of the FEL is that it employs feedback error as a learning signal, which is fundamentally distinctive in the control literature.

This study describes a shared control scheme based on a fuzzy logic approach for a brain-controlled mobile robotic system to improve its performance, safety, and robustness. In contrast to conventional controllers—such as state-of-the-art control methods like SMC and MPC [28,29]—the SLNF controller does not need an accurate mathematical model, which can be challenging to obtain in real-world robotics applications. Furthermore, the SLNF controller deviates from traditional fuzzy controllers by not requiring offline training or prior knowledge. Hence, it can learn and update its parameters online; this feature makes it great for dynamic systems and helps to minimize the disturbance impact.

The contributions of this work are twofold: firstly, this paper discusses the implications of applying an SLNF control technique to a brain-controlled robotic system—a novel approach that has been unexplored previously in this field. Second, it highlights the advantages of the SLNF controller: model-free control, the ability to learn and adapt online, and the capability to minimize the disturbance impact. These insights significantly enrich our understanding of how the SLNF control technique enhances the performance, adaptability, and robustness of a brain-controlled robotic system.

The remainder of this paper is organized as follows: The architecture of the brain-controlled mobile robotic system is illustrated in Section 2. Section 3 demonstrates the controller design of the proposed shared control system. Section 4 describes some key experimental results, while Section 5 concludes the discussion with some potential future directions.

## 2. System Structure

**Figure 1:**
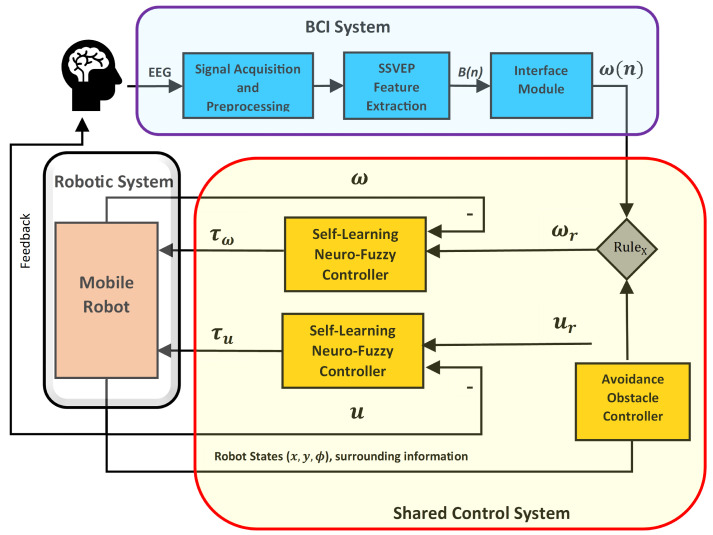
Schematic of the proposed methodology for an EEG controlled mobile robot. The BCI system translates human intentions into steering commands. The shared control system manages automatic switching between user input and autonomous navigation, following ${\mathrm{Rule}}_{X}$ to ensure safety. The robotics system communicates the robot’s states and surrounding information among different controller nodes.

The structure of the proposed control system is illustrated in Figure 1. It contains three subsystems: the BCI system, the robot, and its shared control system, which includes the SLNF controllers and an obstacle avoidance Controller (OAC). Whenever the user intends to drive the mobile robot—based on the surrounding information and robot states—the EEG signals are communicated to the BCI system. The purpose of the BCI system is to translate human intentions into steering commands. There are three parts of the BCI system. In the first part, EEG signals are captured from users and some preprocessing (e.g., noise removal, filtering, etc.) is performed. The second part extracts the important features (e.g., frequency domain features) and categorizes them into three mental states. Finally, the interface module converts these mental states into steering commands (i.e., moving forward, left/right turns). This paper has considered only angular velocity as a steering command. There are two simultaneous commands: one from the BCI system (subject), and the other from the OAC. The shared control system compares these commands and navigates the robot following $Rule_{X}$, taking the environmental situation into account. More details about shared control are reported in Section 2.3. The proposed SLNF controllers ultimately produce the actuator torques (i.e., $\tau_{u}$) required to drive the mobile robot. Note that the human is continuously in the loop.

$Rule_{X}$: If a robot is in a safe state (i.e., not colliding with obstacles and walls), then the output of the BCI system is taken as the reference angular velocity ($\omega_{r}$) for the SLNF controller. Conversely, if a robot is not in a safe state, the output of the OAC is taken as the reference angular velocity.

### 2.1. SSVEP BCI Module

In our work, we used the steady-state visually evoked potential (SSVEP) BCI interface to produce EEG signals from visual stimuli [40]. The SSVEP visual stimuli were displayed on a screen, which comprised two flashing rectangle checkerboards at 12 Hz and 13 Hz, respectively. To control the mobile robot in the corresponding direction (i.e., right or left), a user must concentrate on the corresponding checkerboards (i.e., right or left). On the other hand, when a user intends to keep the robot in the current heading direction (going forward), he or she does not need to attend to any stimulus. EEG signals were captured with an EMOTIV EPOC+ 14-Channel Wireless EEG Headset (at a sampling rate of 2048 Hz) and preprocessed by using a high-pass IIR filter (0.16 Hz) to remove the DC offset. EEG sensors are located at standard positions of AF3, F7, F3, FC5, T7, P7, O1, O2, P8, T8, FC6, F4, F8, and AF4 locations based on the 10-20 system (EEG), while P3/P4 locations (left/right mastoid of the temporal bone) are taken as the electrical reference point and the noise cancellation electrode. To extract the EEG epoch as samples, we adjusted the window length up to 4 s with the step size of 0.5 s. The frequency-domain features were extracted using the discrete wavelet transform (DWT) [41]. Each EEG signal was decomposed into five levels using Daubechies wavelets (db8). As a result, we attained five features set for each channel. Therefore, we got a total of 14 × 5 = 70 features. Support vector machine (SVM) with a one-vs-all classification technique was used to build the recognition model for different mental commands: turning right, turning left, and moving forward.

The interface module is utilized to translate mental commands ω(n) into the reference angular velocity ($\omega_{r}$) (i.e., steering control command):

(1) $$\begin{array}{c} \omega \left(n\right)=\left\{\begin{array}{c} \min\{\omega \left(n-1\right)+\Delta \omega \times B\left(n\right),\omega_{\max}\},B\left(n\right)=1,\\ \omega (n-1),\;B(n)=0,\\ \max\{\omega \left(n-1\right)+\Delta \omega \times B\left(n\right),-\omega_{\max}\},B\left(n\right)=-1 \end{array}\right. \end{array}$$

where ω(n) indicates the angular velocity at nth update, $\omega_{min}$, and $\omega_{max}$ are the minimum/maximum values of the angular velocities respectively,

B(n)=1: turning left,

B(n)=0: moving forward,

B(n)=−1: turning right.

Δω was set to 0.35rad/s, which can be further adjusted. Further details about the BCI module can be found in [40].

### 2.2. Mobile Robot Model

**Figure 2:**
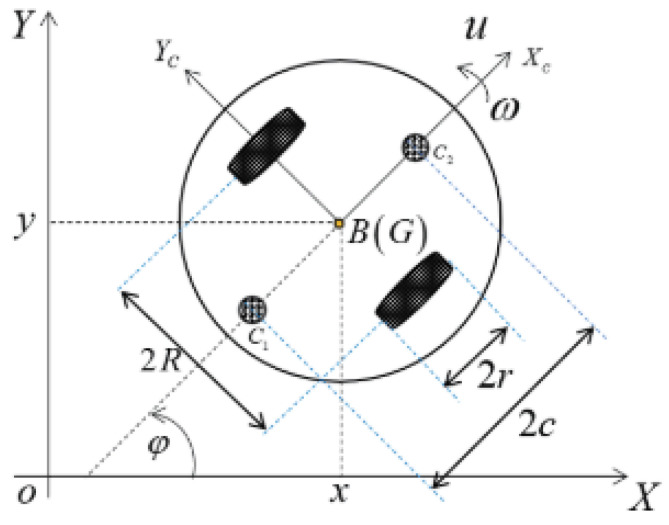
Schematic of the wheeled mobile robot showing a two-dimensional coordinate system with the x−o−y global frame and the $x_{c}-B-y_{c}$ local frame of the robot. G represents the center of gravity of the robot. The distance between the wheels is 2R, and the diameter of each wheel is 2r. ω and *u* represent the angular and linear velocities of the robot, respectively. The angle ϕ indicates the rotation between the global and local frames.

Although the mathematical model of a plant to be controlled is not required for designing fuzzy logic controllers—it is used in our work to test the control performance of the proposed control system with the help of a simulated robot. We considered a wheeled mobile robot (WMR) with two caster wheels and two driving wheels, as shown in Figure 2 [28]. The local and global coordinate systems are indicated as $x_{c}-B-y_{c}$ and x−o−y, respectively (as shown in Figure 2).

The kinematic model of a WMR is represented as (2):

(2) $$\dot{q}=S\left(q\right)\zeta$$

In matrix form,

(3) $$\left[\begin{array}{c} \dot{x}\\ \dot{y}\\ \dot{\phi } \end{array}\right]=\left[\begin{array}{cc} \cos\phi & 0\\ \sin\phi & 0\\ 0 & 1 \end{array}\right]\left[\begin{array}{c} u\\ \omega \end{array}\right]$$

where

$q=\left[\begin{array}{ccc} x & y & \phi \end{array}\right]$ represents the real-time position and orientation of the robot local frame in $x_{c}-B-y_{c}$, and $\zeta ={\left[\begin{array}{cc} u & \omega \end{array}\right]}^{T}$,

*u* and ω: the linear and angular velocities of the mass center point G,

S(q): the Jacobian matrix of the system used for translating generalized coordinates to workspace variables. The dynamics of the robot in matrix form is given as follows:

(4) $$M\left(q\right)\dot{\zeta }+C\left(q,\dot{q}\right)\zeta =B\left(q\right)\tau +\tau_{d}$$

where

$\tau ={\left[\begin{array}{cc} \tau_{r}-\tau_{l} & \tau_{r}+\tau_{l} \end{array}\right]}^{T}$: are the corresponding actuator torques,

$\tau_{d}$: is the unknown disturbance, including parameter uncertainty.

M(q): represents the positive definite system’s inertia matrix,

$C(q,\dot{q})$: is the Coriolis matrix,

B(q): denotes the input transformation matrix.

These matrices are defined as follows:

(5) $$\begin{array}{cc} M(q) & =\left[\begin{array}{cc} \frac{2I_{m}}{r}+mr & 0\\ 0 & \frac{2R}{r}I_{m}+\frac{r}{R}I \end{array}\right]\\ C(q,\dot{q}) & =\left[\begin{array}{cc} \frac{2B_{m}}{r} & 0\\ 0 & \frac{2RB_{m}}{r} \end{array}\right]\\ B(q) & =\left[\begin{array}{cc} 1 & 0\\ 0 & 1 \end{array}\right] \end{array}$$

where *m*, *r*, 2R denote the robot mass, the robot radius, and the lateral distance between the driving wheels, respectively. $I_{m}$ represents the effective moment of inertia (MoI) and the viscous friction coefficient of the motor rotor, *I* is the moment of inertia, and $B_{m}$ denotes the gearbox and wheel assembly. For further details and derivations of the mobile robot model, see [28,42]. The dynamic model of the mobile robot, simplified by reference [28], can be expressed by (6):

(6) $$\left[\begin{array}{c} \dot{u}\\ \dot{\omega } \end{array}\right]=\left[\begin{array}{cc} -\frac{2B_{m}}{\lambda_{u}} & 0\\ 0 & -\frac{d^{2}B_{m}}{\lambda_{\omega }} \end{array}\right]\left[\begin{array}{c} u\\ \omega \end{array}\right]+\left[\begin{array}{cc} 0 & \frac{r}{\lambda_{u}}\\ \frac{dr}{\lambda_{\omega }} & 0 \end{array}\right]\left[\begin{array}{c} \tau_{r}-\tau_{l}\\ \tau_{r}+\tau_{l} \end{array}\right]+\tau_{d}$$

where $\lambda_{u}=\left(m+\Delta m\right)r^{2}+2I_{e}$, $\lambda_{\omega }=2\left(I_{z}+\Delta I_{z}\right)r^{2}+d^{2}I_{e}$, d=2R, $I_{e}$ is the motor inertia, and $I_{z}$ is the inertia of the mobile robot.

### 2.3. Shared Control

The concept of shared control is useful when a system is under the supervision of both a human operator and an embedded intelligent system, such as a robot—where the goal is to assist the users in the navigation of the robot when they are incapable of performing specific maneuvers independently and safely. In default mode, the robot goes forward at a constant speed; it turns left or right after receiving mental commands from users. Users are given complete control over the robot if they do not need any navigational assistance to achieve their goals. In any other case, the system interrupts the mental commands from the user and drives the robot autonomously.

There are two main reasons why shared control is useful when controlling the robot using BCI. Firstly, mental commands from the user (i.e., BCI output) are not always perfect due to user error or fatigue, thereby the robot needs extra navigational safety. Second, there are three possible steering commands (i.e., Forward, Left turn, Right turn) in our study; consequently, the system needs to provide some assistance for fine maneuvering. The shared control system has three controllers, including two SLNFs and one OAC. Details about these controllers can be found in Section 3.

## 3. Controller Design

In the case of brain-controlled mobile robots, direction control is considered more often and with greater importance than speed control. In our study, we employed a kinematic model to predict the position of the mobile robot while considering its velocity. Accordingly, two SLNF controllers are developed for tracking the velocities: one for angular velocity ($\omega_{r}$), reflecting human intentions, and another for linear velocity ($u_{r}$)—maintained at a constant value. In addition, an obstacle avoidance controller is also being developed to guarantee the safety of the robot. The SLNF controller learns and updates its parameters online without prior knowledge or training data. Another key role of the SLNF controller is to deal with disturbances and lessen their effect over time, as demonstrated in Section 4.3.

### 3.1. Self-Learning Neuro-Fuzzy Controller

**Figure 3:**
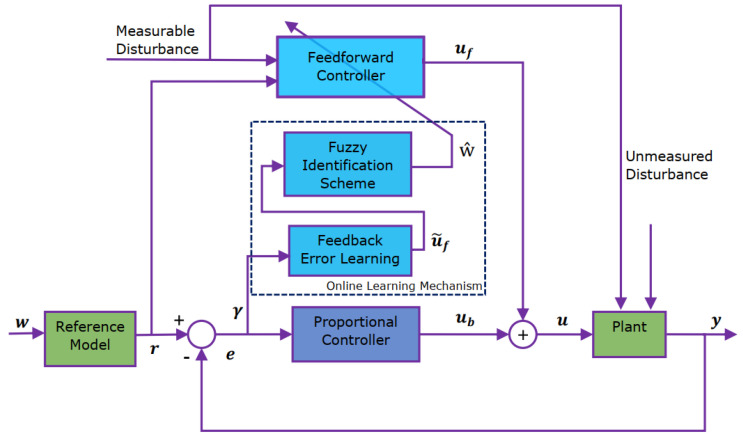
Framework of the self−learning neuro−fuzzy control scheme. The reference model filters the desired changes in the plant’s output (w), guiding the plant to follow the set−point trajectory (*r*). The proportional feedback controller (with $k_{p}$ gain) minimizes the impact of unmeasured disturbances. The feedback error learning module estimates the correct control signal (${\tilde{u}}_{f}$), while the fuzzy identification scheme updates the controller parameters (ŵ). The feedforward controller (neuro−fuzzy model) approximates the inverse model of a nonlinear plant when properly trained.

The general framework of the SLNF controller is represented in Figure 3 [38,43]. It comprises of a feed-forward controller, an online learning mechanism, a reference model, and a proportional controller. A neuro-fuzzy model—a fuzzy system designed consistent with the configuration of a neural network—is utilized to build the controller in the feed-forward path. It combines the learning capacity of neural networks with the linguistic reasoning of fuzzy models.

The feed-forward controller (neuro-fuzzy model) is likely to resemble the inverse model of a non-linear plant once correctly trained, y(t)=f{y(t−1),…,u(t−L),u(t−L−1),…}, where *L* denotes the transportation delay, which is the integral value of sampling time. Since the desired control action is not known at the beginning, thus, online learning is made possible through feedback error learning law [39] as shown in the following relation:

(7) $${\tilde{u}}_{f}=u_{f}\left(t-t_{d}\right)+\gamma e\left(t\right)$$

$u_{f}\left(t-t_{d}\right)$: the inaccurate control action produced $t_{d}$ samples ago,

$t_{d}=L+1$: the delay resulting from the dead-time of plant model,

e(t): the system error, and

γ: the feedback error learning rate.

The online learning mechanism includes two approaches: feedback error learning and fuzzy identification.

#### 3.1.1. Feedback Error Learning

The function of the feedback error learning module is to estimate the correct control signal (${\tilde{u}}_{f}$). Subsequently, the fuzzy identification scheme will utilize this correct control signal to update the correct controller parameters (ŵ).

#### 3.1.2. Fuzzy Identification Scheme

The fuzzy identification scheme is employed in combination with the Fuzzy Least Mean Square (FLMS) Algorithm [38]. This scheme is used in conjunction with the Normalized Least Mean Square Algorithm (NLMS) update rule—as it is computationally effortless [44]. This scheme provides the updated controller parameters ŵ.

#### 3.1.3. Fuzzy Feedforward Controller

Consider a neuro-fuzzy model containing *n* inputs $\left(x_{1},x_{2},\ldots ,x_{n}\right)$ and a single output ($u_{f}$) where the ith input space is divided into $p_{i}$ triangular fuzzy sets with 50% overlap. The Takagi-Sugeno-Kang (TSK) fuzzy inference system has the following $p=\prod_{j=1}^{n}p_{j}$ rules:

*Rule i*: if $x_{1}$ is $A_{11}$, $x_{2}$ is $A_{21},\ldots ,$ and $x_{n}$ is $A_{n1}$, then $u_{f}={\hat{w}}_{1}$.

*Rule*ii: if $x_{1}$ is $A_{11}$, $x_{2}$ is $A_{21},\ldots ,$ and $x_{n}$ is $A_{n2}$, then $u_{f}={\hat{w}}_{2}$,

⋮

*Rulep*: if $x_{1}$ is $A_{1p_{1}}$, $x_{2}$ is $A_{2p_{2}},\ldots ,$ and $x_{n}$ is $A_{np_{n}}$, then $u_{f}={\hat{w}}_{p}$.

Using the multiplication operator and algebraic addition to implement logical ANDs, logical ORs, and height defuzzification, the output of the neuro-fuzzy model [45] can be written as:

(8) $$\begin{array}{cc} u_{f}\left(t\right) & =\sum_{i=1}^{p}a_{i}\left(x\left(t\right)\right){\hat{w}}_{i}\\ \; & =a^{T}\left(t\right)\hat{w}\left(t\right) \end{array}$$

where

$a_{i}\left(x\left(t\right)\right)$: the product of the membership grades in the fuzzy sets in the antecedent part of the ith rule,

$x\left(t\right)={\left[x_{1}\left(t\right)\cdot x_{2}\left(t\right)\ldots x_{n}\left(t\right)\right]}^{T}$ is Kronecker tensor product of the input vector,

$a\left(t\right)=\left[a_{1}\;a_{2}\ldots a_{p}\right]$ is its transformed vector,

$A_{i}$ denotes ith fuzzy set.

$\hat{w}\left(t\right)={\left[\hat{w}{\left(t\right)}_{1}\left(t\right)+\hat{w}{\left(t\right)}_{2}\left(t\right)+\ldots +\hat{w}{\left(t\right)}_{p}\left(t\right)\right]}^{T}$ is a weight vector of the controller’s parameters.

The main objective is to determine the elements in the weight vector (ŵ) to facilitate the mapping of the input vector to the control signal by the feedforward controller. The parameters ŵ of feedforward neuro-fuzzy controller can be estimated at a time interval, by sending the data pair $x\left(t-t_{d}\right),{\tilde{u}}_{f}$ to the fuzzy identification scheme, which can be found by the following expressions:

(9) $$\hat{w}\left(t\right)=\hat{w}\left(t-1\right)+\delta \frac{S\left(t-1\right)a\left(t-t_{d}\right)}{a^{T}\left(t-t_{d}\right)S\left(t-1\right)a\left(t-t_{d}\right)}\varepsilon \left(t\right)$$

δ: the user-selected update rate, and

ε(t): the modeling error, whereas all elements in $\hat{w}\left(t-1\right)$ are initialized at zero.

(10a) $$\begin{array}{cc} S(t) & =\mathrm{diag}\{s_{1},s_{2},s_{3},\ldots ,s_{i},\ldots ,s_{p}\} \end{array}$$

(10b) $$\begin{array}{cc} s_{i} & =\min\left\{\prod_{j=1,j\neq i}^{p}F_{j}\left(t\right)\right\} \end{array}$$

(10c) $$\begin{array}{cc} F_{i}\left(t\right) & =F_{i}\left(t-1\right)+a_{i}\left(t\right) \end{array}$$

where S(t) is a diagonal matrix that represents the cumulative strength at which a rule has been fired, while all elements in S(t−1) are initialized at unity. $F_{i}\left(t\right)$ determines the ith rule’s firing rate and strength. It is initialized at unity and has a maximum bound of 1000. It also indicates the accuracy of parameters in the ŵ(t) weight vector.

(10d) $$\varepsilon \left(t\right)={\tilde{u}}_{f}\left(t\right)-a^{T}\left(t-t_{d}\right)-\hat{w}\left(t-1\right)$$

The total control action is given as:

(11) $$u\left(t\right)=u_{f}\left(t\right)+k_{p}e\left(t\right)$$

#### 3.1.4. Proportional Controller

The purpose of incorporating a proportional feedback controller (with $k_{p}$ gain) is to lower the impact of any unmeasured disturbances while ensuring satisfactory control performance. The controller has no prior knowledge of the plant to be controlled initially; thereby, it cannot generate the correct control signal because its parameters have not been learned yet.

#### 3.1.5. Reference Model

The main idea of using this block is to filter out the required changes in the output of the plant (w). The plant follows the set-point trajectory (*r*) provided by the reference model. Theoretically, a plant with a feed-forward controller should imitate the reference model behavior.

### 3.2. Obstacle Avoidance Controller (OAC)

**Figure 4:**
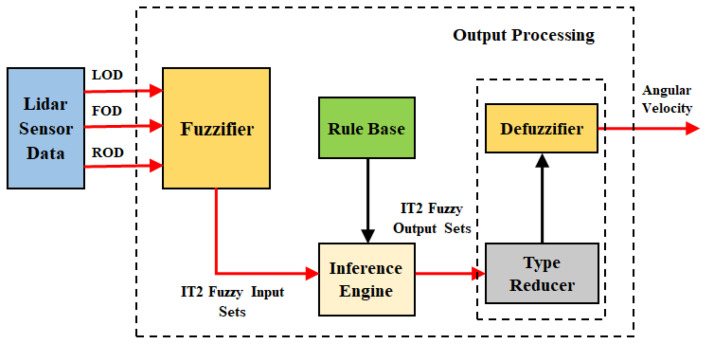
Structure of Obstacle Avoidance Controller (OAC). IT2FLS processes three LIDAR distance inputs, fuzzifies them into IT2 fuzzy sets, and applies inference rules. The type reducer converts these to IT1 fuzzy sets, and the defuzzifier computes the angular velocity for robot control.

We used three LIDAR sensors—which can be further extended to 5 or 7—to design the membership functions (MFs) of OAC. These MFs are designed based on the Interval Type-2 Fuzzy Logic System (IT2FLS) scheme, by using LIDAR sensor data collected from the mobile robot. The framework of the IT2FL system is presented in Figure 4. The primary function of OAC is to move the mobile robot away from the boundary walls and obstacles, avoiding potential collisions. It is responsible for both environmental and operational safety.

Compared to the Type-1 Fuzzy Logic System (T1FLS), IT2FLS has an extra output processing block, which includes type reduction followed by the defuzzifier block [46]. The type reduction block maps IT2FLS into T1FLS, and afterward, the defuzzifier maps that T1FLS into a crisp angular velocity. The structure of the rules in IT2FLS remains the same as in T1FLS. There are two major architectures for an IT2FLS: Mamdani and TSK. We used the Mamdani type in OAC because it is very suitable to human cognition [47,48,49]. The IT2 Mamdani type has all of the antecedent and consequent MFs in IT2FLS which offers more flexibility to handle uncertainties due to its adjustable parameters, and offers easy representation of uncertainities [46,50,51].

**Figure 5:**
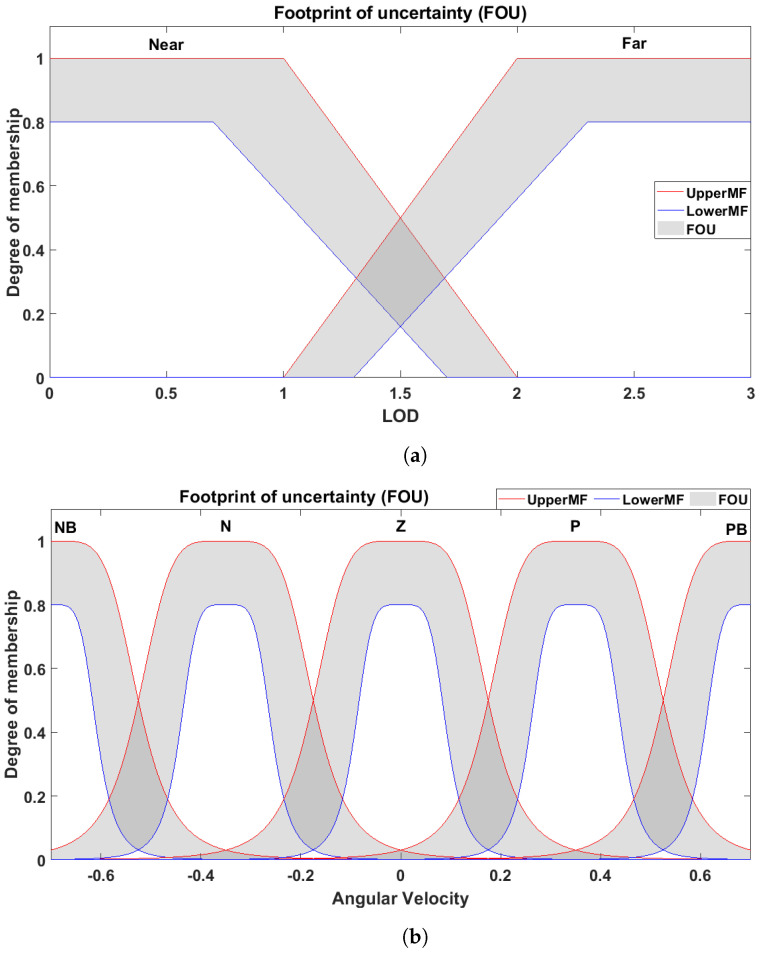
The membership functions of AOC for (**a**) input variables and (**b**) output variables; Note: LOD = FOD = ROD.

The IT2FLS receives three crisp inputs from the LIDAR sensors—representing distances (adjustable within a range of up to 5 m) to the nearest obstacle (i.e., LOD, FOD, and ROD)—and provides the angular velocity as an output to the robot. LIDAR sensors were configured as follows: one is mounted at an angle of π/2 on the vertical axis, and the other two were mounted at angles of −π/4 and π/4 with reference to the vertical axis. We denoted each input by two IT2 MFs, Near and Far, whereas we used five output MFs for the OAC, as shown in Figure 5. The rules for OAC are determined using expert knowledge of the operator and are listed in Table 1. The linguistic terms used in OAC have the following meanings: LOD, FOD, and ROD are the left, forward, and right distances, respectively. P, PB, Z, N, and NB denote Positive, Positive Big, No Turn, Negative, and Negative Big, respectively.

## 4. Results

To evaluate the control performance of the SLNF controller developed in Section 3, we conducted human-in-loop (HIL) simulation experiments with human subjects controlling a simulated mobile robot. Eight subjects participated in the experiment without monetary compensation. None of the subjects had a history of neurological or mental illness, and none had completed the navigation of a brain-controlled robot. This study adhered to the guidelines of the 2013 Declaration of Helsinki. Informed consent forms were signed by all the participants.

### 4.1. Simulation Setup, and Test Scenario

Only subjects who demonstrated a high level of accuracy in the SSVEP BCI—defined as a performance threshold greater than 90%—were included in the study. Accordingly, subject eight is excluded from the analysis.

As detailed in Section 2, the architecture of the brain-controlled robotic system comprises three principal components: a shared control system, a BCI system, and a robotic subsystem. Transmission Control Protocol/ Internet Protocol communication (TCP/IP) is used to transmit data between the BCI system and the mobile robot.

The robotic control system is executed using MATLAB R2020b software. The physical parameters of the robot dynamics used in our simulation were as follows: $m\;=\;16.5\;\mathrm{kg},\;r\;=\;0.0625\;m,\;d\;=\;0.340\;m,\;B_{m}\;=\;0.1,\;I_{z}\;=\;0.68\;\mathrm{kg}\;\cdot \;m^{2},\;I_{e}\;=\;0.0015\;\mathrm{kg}\;\cdot \;m^{2}.$

Nine triangular MFs for the reference input with 50% overlap equally spaced between the ranges (0, 1) were used in the SLNF controllers. The SLNF controller’s user-selected parameters can be determined from a discrete PI controller, as explained in [38]. By setting the update rate to unity (δ=1), the learning rates and feedback proportional gains of the SLNF controllers for the linear and angular velocities were adjusted to $\gamma =0.3,\;k_{p}=1.5,\;\mathrm{and}\;\gamma =0.85,\;k_{p}=\;2.8.$, respectively. Details of the Fuzzy-PID controller can be found in our previous work [34].

**Figure 6:**
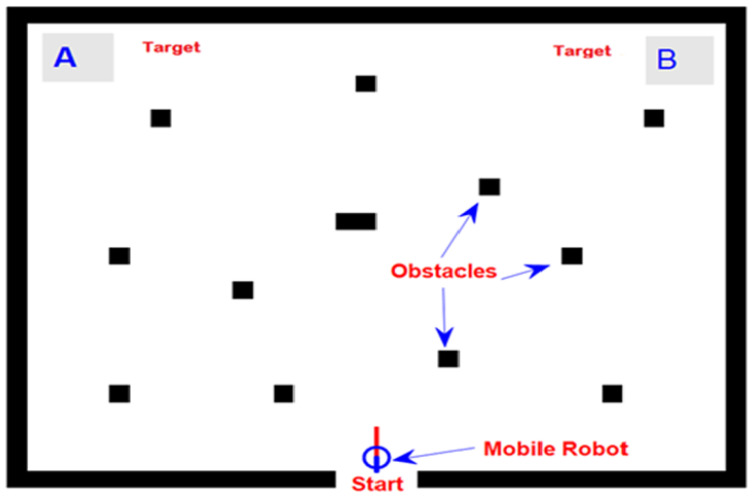
Online simulation setup of a robotic system where a user maneuvers the mobile robot using EEG signals to targets A or B, avoiding obstacles.

Figure 6 shows an indoor environment of a simulated mobile robot, in which four black boundary stripes form a rectangular area that represents the safe navigation region for a mobile robot. In Figure 6, the blue hollow circle indicates a simulated mobile robot, and the red line points to the current heading direction of the mobile robot to help the subject navigate it through a cluttered environment. Moreover, the operating region contained many randomly scattered black rectangular boxes that represented detectable static obstacles.

The subjects were given the task of controlling a mobile robot from the start to one of the two target positions (A or B) by attending to related SSVEP stimuli. The online brain-controlled HIL simulation required the subjects to perform ten runs per controller type. The robot is expected to take the minimum amount of time possible while attempting to avoid collisions with any obstacles. The default position for the robot is set as the starting location at the beginning of the simulation. In our simulation test scenario, we assumed that the robot could pass through obstacles; however, once it crossed the boundary region, the trial was considered terminated. We evaluated the performance of the robot control system using different control methods: Direct BCI control, Fuzzy-PID control, and the proposed SLNF controller. Direct BCI control refers to the control of a robot without using the proposed control system.

**Figure 7:**
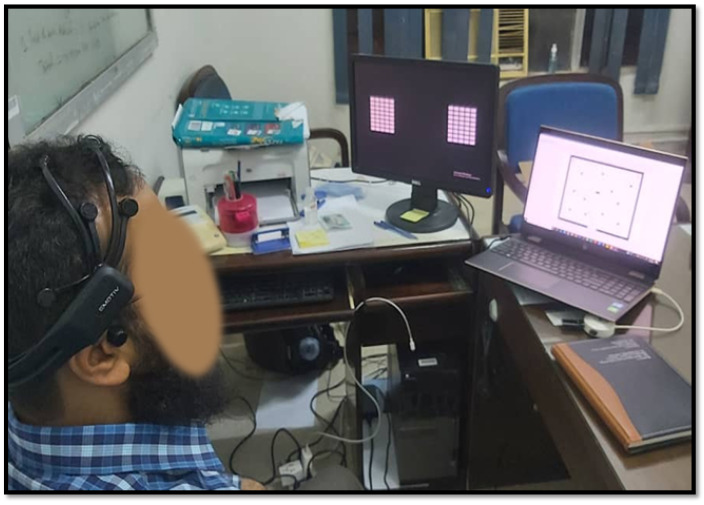
Experimental scenario. The subject focuses on the SSVEP visual stimuli (left screen) to maneuver the robot through obstacles (right screen) and reach the target safely.

Before experimenting, the participants were acquainted with the experimental procedure. Offline training is used to identify BCI module parameters in brain-controlled robot experiments. EEG data is collected for three control commands (turning right, turning left, and going forward) using an EMOTIV EPOC+ wireless headset. Figure 7 illustrates the experimental setup for conducting brain-controlled mobile robot experiments. Subjects were asked to complete 4 sessions of the respective SSVEP stimuli for turning left and right commands every 12 seconds (s) while not attending to any stimuli for going forward commands. Each session comprised four trials, and the subjects took a 20 s break between two consecutive trials. SSVEP BCI Accuracy values for different subjects are reported in Table 2. The accuracy metric for each control command can be calculated as:

$$\mathrm{Accuracy}=\left(\frac{\mathrm{No}.\;\mathrm{of}\;\mathrm{correctly}\;\mathrm{classified}\;\mathrm{commands}}{\mathrm{Total}\;\mathrm{number}\;\mathrm{of}\;\mathrm{commands}}\right)\times 100\%$$

### 4.2. System Performance Evaluation

We used three metrics [28]—task completion time, task completion rate, and total collisions—to assess the control system performance of the proposed control method against direct BCI control and Fuzzy-PID control methods.

As per definition, the task completion rate is the number of successful trials divided by the total number of trials. The nominal task time ($T_{nm}$) is calculated as the task completion time using the shortest distance between the target point and the starting point with the desired linear velocity of the robot [40]. In our experiments, a trial is considered successful if the subject is capable of guiding the robot to the desired location; whereas the task completion time is assumed to be less than three fold of the nominal task time ($3\times T_{nm}$). Finally, the total number of collisions is defined as the number of collisions encountered during each trial.

**Figure 8:**
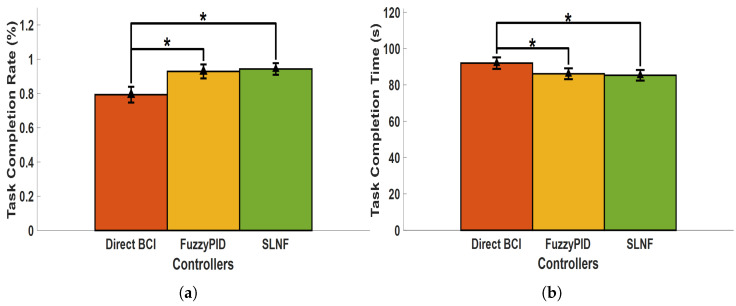
Comparison of system performance among the Direct BCI, Fuzzy-PID, and SLNF controllers for (**a**) Task completion rate (%) and (**b**) Task completion time (seconds). The SLNF controller achieved a higher average task completion rate of 94.29% (vs. 79.29% for Direct BCI and 92.86% for Fuzzy-PID) and a shorter average task completion time of 85.31 s (vs. 92.01 s for Direct BCI and 86.16 s for Fuzzy-PID). Statistically significant differences are indicated with (*) for *p* < 0.001.

Results were analyzed using a statistical analysis, involving a one-factorial ANOVA with Bonferroni-adjusted post-hoc tests. Figure 8 illustrates the system performance for the different control methods (BCI, Fuzzy-PID, and SLNF), including means and standard errors, highlighting significant findings. Figure 8a depicts the mean task completion rate along with standard errors. We observed a significant difference in task completion rate among controllers (F(1,12)=17.779, p<0.001, partial $\eta^{2}=0.748$). The SLNF controller significantly outperformed the direct BCI control method (p=0.008) by 15% (94.29% ± 3.4% vs. 79.29% ± 4.6%), whereby no significant differences were observed for the Fuzzy-PID controller (94.29% ± 3.4% vs. 92.86% ± 4.1%). Figure 8b presents the average task completion times along with respective standard errors. We observed a significant difference in task completion time among controllers (F(1,12)=71.867, p<0.001, partial $\eta^{2}=0.923$). The SLNF controller exhibited a significantly shorter average task completion time (p<0.001) of 85.309 ± 2.926 s, in contrast to the Direct BCI control method (92.011 ± 3.167 s), whereby descriptively similar performance is observed for the Fuzzy-PID control (86.157 ± 2.997 s). Our SLNF controller reduced mean task completion time by 6.70 s compared to Direct BCI and was 0.85 s faster than the Fuzzy-PID controller.

**Figure 9:**
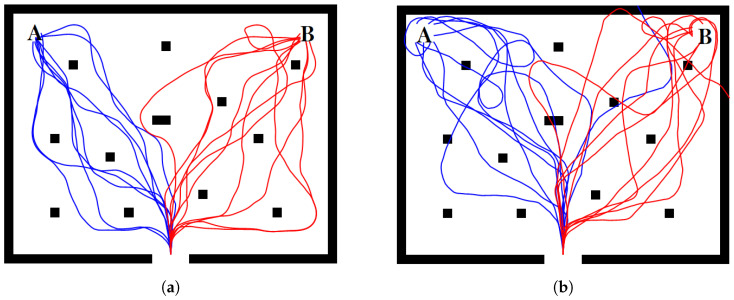
Robot trajectories produced by Subject Three using (**a**) the proposed SLNF controller and (**b**) Direct BCI control. The trajectories with the proposed SLNF controller show no collisions, demonstrating the efficacy of OAC, while Direct BCI control method was unable to handle obstacle avoidance.

Both the proposed SLNF and Fuzzy-PID controllers successfully navigated the robot without any collisions; whereas the direct BCI control method had a mean collision number of 6.650 with standard errors of ±1.304. Figure 9 further shows the recorded trajectories of the mobile robot for subject Three during tasks A and B. Results indicate much smoother trajectories with fewer collisions for the proposed SLNF controller compared to direct BCI control.

### 4.3. Robustness Evaluation

**Figure 10:**
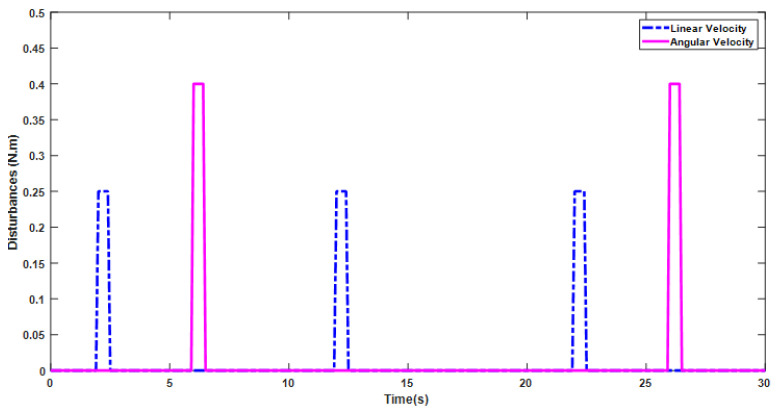
Step input disturbance torque signals. These test the disturbance handling capability of our proposed control system.

Another important feature of the SLNF controller is its ability to overcome the effects of disturbances over time. In our work, two pulse signals were introduced as input torque disturbances to check the robustness of our proposed control system. These simulated signals have amplitudes of 0.25 Nm and 0.4 Nm and periods of 10 s and 20 s for linear and angular velocities, respectively, as shown in Figure 10.

**Figure 11:**
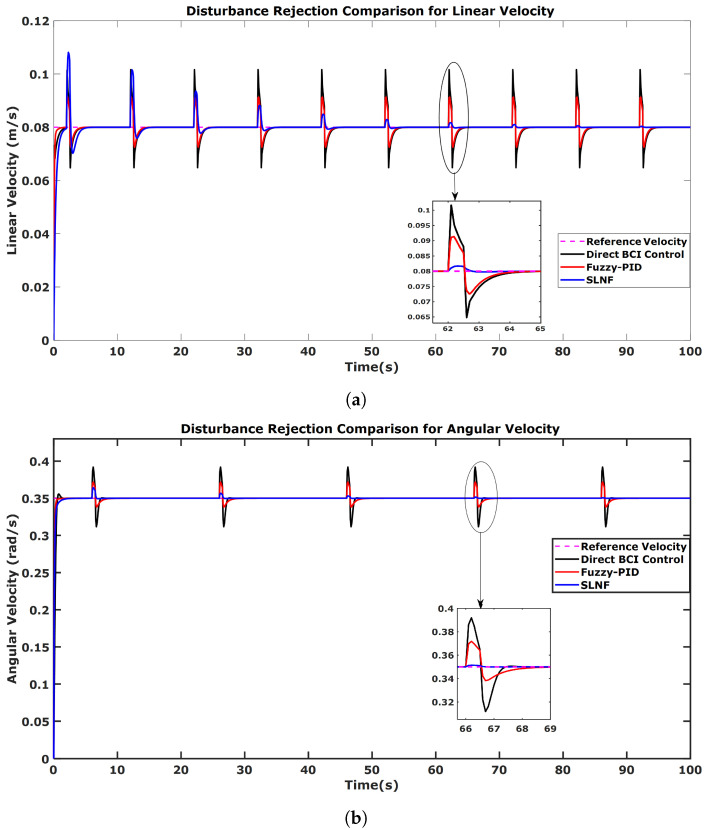
Disturbance rejection comparison among Direct BCI control, fuzzy-PID, and the proposed controller for (**a**) linear velocity and (**b**) angular velocity. Initially, the SLNF controller exhibits the highest overshoot in linear velocity but reduces it over time due to its online learning capability, as shown in the zoomed area. For angular velocity, the SLNF controller shows minimal overshoot and settling time, with disturbance effects decreasing over time. Direct BCI and Fuzzy-PID controllers do not show a significant reduction in disturbance.

The linear velocity ($u_{r}$) is kept constant in our control system at 0.12 m/s; whereas the angular velocity ($\omega_{r}$) keeps changing to control the direction of the mobile robot. We provided step inputs to our system to better understand the characteristics and disturbance-handling ability of our proposed control method, as shown in Figure 10. Good tracking abilities were observed for all control schemes (e.g., direct BCI, fuzzy-PID, and SLNF) in the absence of simulated disturbances; however, in the case of disturbances (spikes in Figure 11), the two control methods, Direct BCI control and fuzzy-PID, didn’t perform well as they could not overcome the disturbance effect as shown in the zoomed areas of Figure 11. On the contrary, the proposed SLNF controller managed to successfully minimize the effect of disturbances with time—despite initially higher overshoots (as shown in the zoom box of Figure 11a since it can update its parameters online. Thus, once trained, the proposed SLNF controller outperforms the other control methods (direct BCI control and Fuzzy-PID) in terms of overshoot and settling time, see Figure 11b. Moreover, the SLNF controller mitigated the effect of disturbance over time, whereas direct BCI control failed to do it.

To summarize, the Direct BCI control method could not handle the effect of disturbances; while the Fuzzy-PID controller partially lessened the effect. Conversely, the proposed SLNF controller successfully dealt with disturbances as time progressed due to its online learning capability. This suggests that the proposed control framework has improved the robustness of the system.

## 5. Conclusions

This paper presents a shared control scheme based on a fuzzy-logic approach to improve the performance of a brain-controlled mobile robot. In other words, we investigated the applicability of our proposed method to a brain-controlled robotic system and discussed the advantages of the SLNF controller over traditional methods. Moreover, we developed two SLNF controllers for tracking the linear and angular velocities of the robot, and one OAC for the safety of the robot. The SLNF controllers—due to their online learning capability—successfully tracked the desired trajectories of a BCI mobile robot and effectively diminished the effects of external disturbances, which depicts the robustness of the system. The shared control technique compared the steering commands from the BCI system with surrounding information to ensure safety. Conversely, if these control commands violated the safety constraints (i.e., due to collisions with walls or obstacles), the shared control system passed the control fully to the robot by overriding the direct BCI commands; consequently, the mobile robot moved autonomously while avoiding obstacles.

The human-in-loop simulation demonstrates the efficacy of the proposed control strategy by achieving: a higher task completion rate, shorter task completion time, and no collisions compared to Direct BCI control and also outperformed the Fuzzy-PID controller in terms of disturbance rejection. Consequently, users can perform a given task safely and robustly. However, it is important to note that no notable difference is observed in the task completion rate or time when comparing our proposed controller with the Fuzzy-PID controller. Perhaps, the SLNF controller is trained based on the PI controller, whereas Fuzzy-PID is trained based on the PID controller; this might have resulted in comparable control performance. Nonetheless, it should be noted that the proposed controller effectively diminished the impact of disturbances over time—highlighting the robustness of our proposed control method. Hence, we conclude that the proposed control approach improves the safety, performance, and robustness of brain-controlled mobile robots.

Although the proposed control method has improved the robotic control system—it has certain limitations. First, we only performed online simulation experiments, and the subject’s experience would be slightly different when controlling a real mobile robot in a cluttered environment. Secondly, we only considered the angular velocity as the steering control command (turn left/right, and keep moving ahead) of the BCI output while maintaining a constant linear velocity.

In future, we will consider dynamic objects and more complex scenarios to further validate the proposed control scheme. For this, another controller may be designed for linear motion control along with the starting and stopping of the mobile robot, although this is a challenging task.

## Figures and Tables

**Table 1:** Rules for AO controller.

Rule	LOD	FOD	ROD	ω
1	Near	Near	Near	Negative Big
2	Near	Near	Far	Negative
3	Near	Far	Near	Zero
4	Near	Far	Far	Negative
5	Far	Near	Near	Positive
6	Far	Near	Far	Negative Big
7	Far	Far	Near	Positive
8	Far	Far	Far	Zero

**Table 2:** Accuracy of SSVEP BCI system (%).

Subject	Turning Left	Turning Right	Going Forward	Mean
One	97.7%	100%	100%	99.2%
Two	97.7%	100%	98.9%	98.9%
Three	97.7%	98.9%	97.7%	98.1%
Four	96.6%	98.9%	97.7%	97.7%
Five	96.6%	94.3%	98.9%	96.6%
Six	93.2%	97.7%	92.0%	94.3%
Seven	98.9%	86.4%	88.6%	91.3%
Eight	81.8%	100%	77.3%	86.4%

## Data Availability

The data used to support the findings of this study is available from the corresponding author upon request.
